# Efficient photocatalytic degradation of textile dye pollutants using thermally exfoliated graphitic carbon nitride (TE–g–C_3_N_4_)

**DOI:** 10.1038/s41598-024-52688-y

**Published:** 2024-01-27

**Authors:** Selvaganapathy Ganesan, Thangavelu Kokulnathan, Shanmugam Sumathi, Arunkumar Palaniappan

**Affiliations:** 1 Department of Chemistry, School of Advanced Sciences, Vellore Institute of Technology, Vellore, Tamil Nadu 632014 India; 2 Human Organ Manufacturing Engineering (HOME), Lab, Centre for Biomaterials, Cellular and Molecular Theranostics (CBCMT), Vellore Institute of Technology, Vellore, Tamil Nadu 632014 India; 3 Department of Electro-Optical Engineering, National Taipei University of Technology, Taipei, 106 Taiwan

**Keywords:** Environmental sciences, Chemistry, Materials science, Nanoscience and technology

## Abstract

Graphitic carbon nitride (g–C_3_N_4_), an organic photocatalyst was reported to have beneficial properties to be used in wastewater treatment applications. However, g–C_3_N_4_, in its bulk form was found to have poor photocatalytic degradation efficiency due to its inherent limitations such as poor specific surface area and fast electron–hole pair recombination rate. In this study, we have tuned the physiochemical properties of bulk g–C_3_N_4_ by direct thermal exfoliation (TE–g–C_3_N_4_) and examined their photocatalytic degradation efficiency against abundant textile dyes such as methylene blue (MB), methyl orange (MO), and rhodamine B (RhB). The degradation efficiencies for MB, MO, and RhB dyes are 92 ± 0.18%, 93 ± 0.31%, and 95 ± 0.4% respectively in 60 min of UV light irradiation. The degradation efficiency increased with an increase in the exfoliation temperature. The prepared catalysts were characterized using FTIR, XRD, FE-SEM, EDAX, BET, and UV-DRS. In BET analysis, TE–g–C_3_N_4_ samples showed improved surface area (48.20 m^2^/g) when compared to the bulk g–C_3_N_4_ (5.03 m^2^/g). Further, the TE–g–C_3_N_4_ had 2.98 times higher adsorption efficiency than the bulk ones. The free radicals scavenging studies revealed that the superoxide radicals played an important role in the photodegradation for dyes, when compared to the hydroxyl radical (^.^OH) and the photo-induced holes (h^+^), Photoluminescence (PL) emission and electrochemical impedance spectroscopy (EIS) spectra of TE–g–C_3_N_4_ indicated a lowered electron–hole pairs’ recombination rate and an increased photo-induced charge transfer respectively. Further, the TE–g–C_3_N_4_ were found to have excellent stability for up to 5 cycles with only a minor decrease in the activity from 92% to 86.2%. These findings proved that TE–g–C_3_N_4_ was an excellent photocatalyst for the removal and degradation of textile dyes from wastewater.

## Introduction

In recent years, globalization, the advancement of technology, coupled with the evolving fashion preferences of humans, has significantly contributed to the augmentation of textile and garment manufacturing^1,2^. Compared to the past decade, consumers buy clothes twice the time and keep them for only half the time^3^. This huge production of textile products and their shorter utilization creates dramatic textile and apparel waste after consumer usage. Also, these industries use synthetic pigments as coloring agents^4–6^. The textile and fashion industries are globally criticized for pernicious environmental impact (water contamination, waste generation, and carbon footprint) throughout their supply chain^7^. For example, The Ganga river in India experiences a daily influx of around 250 million liters of industrial effluent as a consequence of human encroachment in the surrounding regions^8–10^. The pigments used in the textile industries are stable, highly aqueous soluble, toxic, carcinogenic, and non-biodegradable^11,12^. These dyes obstruct the sunlight penetration range and result in reduced photosynthesis process which causes unavailability of oxygen to aquatic organisms. Moreover, the ingestion or inhalation of the toxic dyes is reported to trigger respiratory infections, skin, and eye irritations^13–15^. Also, some dyes are found to get converted into carcinogenic compounds in anaerobic conditions, thus leads to the manifestation of various health conditions, including but not limited to cancer, asthma, diarrhea, nausea, and diverse forms of allergic reactions^16,17^. The most widely used methods for the removal of these hazardous dyes are adsorption, biosorption, filtration, ion exchange, coagulation, and advanced oxidation process (AOP)^18–20^. In recent times, AOP-based photocatalytic wastewater treatment methods have been widely used for environmental depollution due to their advantages such as eco-friendliness, low maintenance cost, easier operation, and high efficiency^21–23^. The photocatalysts are typically semiconductor materials that generate electron–hole pair upon irradiation with light source. This in turn reacts with dissolved oxygen and moisture present in the dye solution to produce reactive ion species. These reactive ion species then convert toxic organic dyes to non-toxic or secondary pollutants^24,25^. Several metal oxides, noble metals, metal carbides, and sulfur-based semiconductors are used as photocatalysts to remove pollutants from the environment^26,27^. However, these metal-based photocatalytic materials have limitations such as high cost, low quantum efficiency, lower availability, and more importantly generation of metallic pollutants when these catalysts are discarded. This leads to a scenario where treatment strategies and components polluting back the environment. Thus, there is a strong need for a metal-free catalyst with better photocatalytic efficiency^28^.

Graphitic carbon nitride (g–C_3_N_4_) is one such metal-free semiconductor with an excellent physico-chemical and photocatalytic properties. The electrons generated through photoexcitation in g–C_3_N_4_ exhibited a higher thermodynamic potential to facilitate the reduction of diverse organic compounds into H_2_O and CO_2_^29,30^. However, g–C_3_N_4_ in its bulk form had several drawbacks, such as a limited specific surface area and an accelerated recombination rate^31,32^ There are reports in the literature where the photocatalytic performance of bulk g–C_3_N_4_ were increased by making composites with metal oxides, quantum dots, and fabricating heterojunction structures^33–37^. However, in the above methods, g–C_3_N_4_ was typically combined with other materials using multi-step reactions, which increased the cost as well as the complexity. Thus, exfoliation of g–C_3_N_4_ using physical methods (e.g. thermal treatment) or chemical methods (e.g. acid etching) was an attractive option to improve their photocatalytic performances, mainly through the enhancement in their surface area due to the formation of this and porous structures. Various researchers have made highly porous and sheet-like g–C_3_N_4_ for enhanced photocatalytic performance through protonated exfoliation or hydrothermal breakdown; however, these procedures were complex and expensive^38–40^. Among them, thermal exfoliation was the most environment-friendly option which was currently being explored.

**Figure 1:**
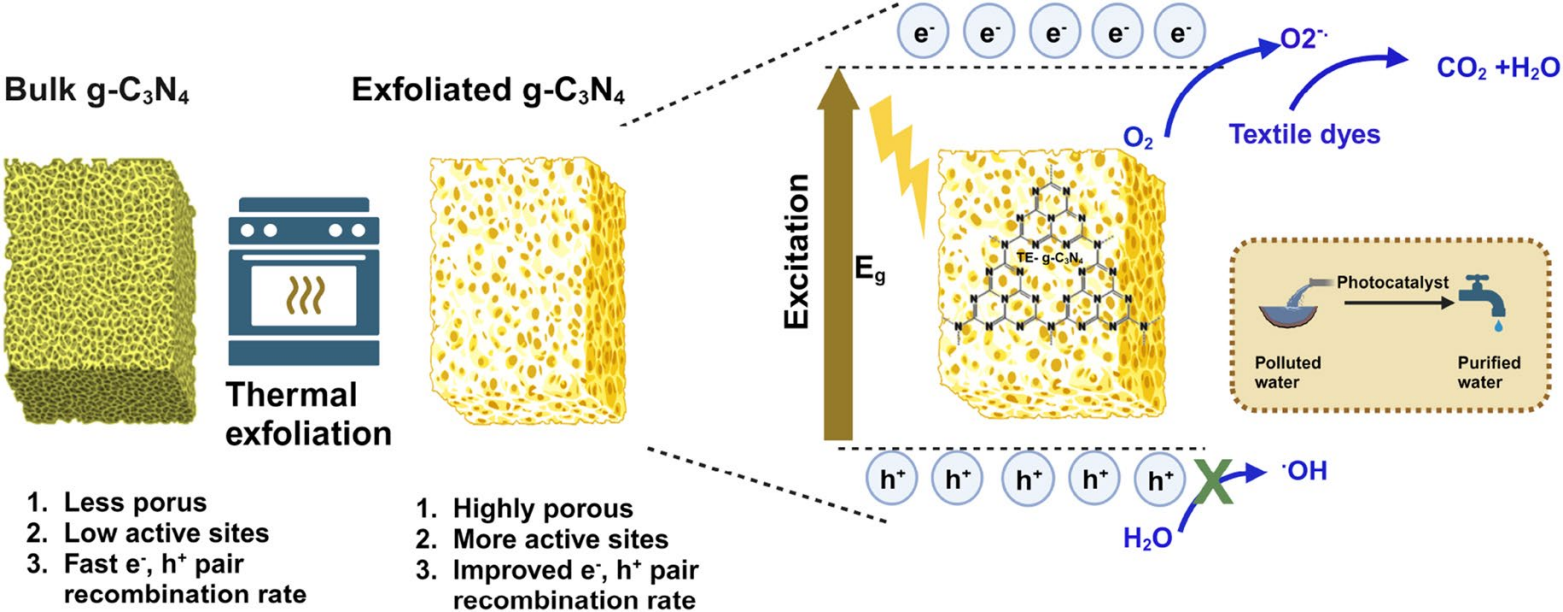
Schematic showing the preparation of TE–g–C_3_N_4_ and its photocatalytic mechanism.

The thermal exfoliation process of g–C_3_N_4_ was a straightforward method for the synthesis of a catalyst that exhibited an excellent porosity and efficiency, making it suitable for many environmental applications (Fig. 1). From the previously published literature, thermal exfoliation of g–C_3_N_4_ was synthesized by various researchers with different precursors and parameters for applications such as NOx removal and H_2_ production^41–44^. In the present work, we reported the preparation of g–C_3_N_4_ by thermal exfoliation at static air and examined its adsorption and photocatalytic degradation efficiency against the most common textile dyes such as methylene blue (MB), methyl orange (MO), and rhodamine B (RhB) dye solutions under UV light irradiations. Finally, we have also investigated the mechanisms through which these thermally exfoliated photocatalysts degraded the dyes using radical scavenging experiments as well as by calculating the band potentials to confirm the active species involved in the reaction. To the best of our current understanding, there is a lack of documented research pertaining to the photodegradation of textile dyes by the utilization of TE–g–C_3_N_4_ using UV light irradiation, which we have addressed through this work.

## Experimental section

### Materials

Melamine (C_3_H_6_N_6_) ≥ 98% was purchased from SD Fine Chemicals Ltd, Mumbai, India. Methylene blue (MB) (C_16_H_18_ClN_3_S), rhodamine B (RhB) (C_28_H_31_ClN_2_O_3_), methyl orange (MO) (C_14_H_14_N_3_NaO_3_S), ammonium oxalate (C_2_H_8_N_2_O_4_) ≥ 99%, and isopropyl alcohol (IPA) (C_3_H_8_O) ≥ 99.5%, potassium chloride (KCL) 99.5%, were purchased from SRL Chemical, Mumbai; para-benzoquinone (P–BQ) (C_6_H_4_O_2_) ≥ 98%, sodium sulfate (Na_2_SO_4_) ≥ 99%, was purchased from Sigma Aldrich, Bengaluru. All chemicals employed in the study were of analytical pure grade and were utilized without undergoing additional purification processes. All studies were conducted using Milli-Q water.

### Synthesis

#### Preparation of bulk g–C_3_N_4_

The bulk g-C_3_N_4_ was synthesized using melamine as per protocol^45^. In a concise manner, a quantity of 10 g of melamine was placed into a sealed crucible made of alumina. The temperature of the crucible was raised to 550 °C at 5 °C per minute. The sample was kept in the above condition for 4 h in the static air, after which samples were cooled down to obtain yellow-colored agglomerates. The agglomerates were then ground to get fine powders using agate mortar.

#### Preparation of TE–g–C_3_N_4_

TE–g–C_3_N_4_ samples were synthesized using the process of heating pre-existing bulk g–C3N4 material within an open alumina crucible, employing various temperature condition of 450 °C, 500 °C, and 550 °C for 2 h with a rate of 5 °C per min. The resultant samples were labelled as g–C_3_N_4_ 450, g–C_3_N_4_ 500, g–C_3_N_4_ 550 corresponding to 450 °C, 500 °C, and 550 °C respectively.

### Characterization

X-ray diffraction (XRD) tests were employed to verify the production and purity of the synthesized materials using Panalytical X’Pert^3^. The characteristic functional groups present in the synthesized materials were identified using Agilent Cary 630 Fourier transform infrared (FTIR) spectroscopy [Agilent Cary 630] studies. The analysis of the samples' surface area was conducted using the Brunauer–Emmett–Teller (BET) technique using Bellsorp Max II analyzer. Surface morphology and elemental analysis of all the materials were examined by field emission scanning electron microscope (FESEM)-Thermo Fisher FEI-Quanta 250 FEG. JASCO V-670 UV–Vis–NIR spectrophotometer was used to obtain the optical properties such as absorbance spectra and band gap. The concentration of the remnant dyes present in the system after a photodegradation experiment was detected using a UV–Vis spectrophotometer (Agilent Cary 3500). Photoluminescence (PL) properties of the materials were investigated with a HITACHI F-7000 fluorescence spectrophotometer.

### Photocatalytic experiments

The photocatalytic activity of the TE–g–C_3_N_4_ samples was investigated using most abundantly used organic textile dyes: MB, RhB, and MO as per the protocol in the literature^46^. Briefly, 0.05% w/v of the TE–g–C_3_N_4_ sample was added into 50 mL dye solutions (10 ppm) and stirred for 30 min in dark conditions to attain adsorption/desorption equilibrium. Subsequently, the samples underwent additional agitation within a photoreactor while being exposed to UV light (with a wavelength of 365 nm and power output of 125 W), in order to assess the catalysts’ ability to degrade the dye. At discrete time intervals, a 3 mL aliquot was extracted from the solution and subjected to centrifugation in order to isolate the catalyst. The amount of dye in the solution was quantified using a UV–Vis spectrophotometer. The adsorption and degradation efficiency of dyes were determined using the absorbance maxima peaks exhibited by the dyes: MB-664 nm, RhB-554 nm, and MO-464 nm in the absence and presence of light irradiation. The radical scavenging studies were performed with the best performing catalyst, g–C_3_N_4_ 550 against all three dyes. The experimental procedure involved the addition of specific trapping agents, namely ammonium oxalate (AO), isopropyl alcohol (IPA), and para-benzoquinone (P–BQ), in order to scavenge the photogenerated holes (h^+^), hydroxyl radicals (^.^OH), and superoxide radicals (O_2_^.^^–^) species, respectively.

The degradation percentage of each dye and the rate constant of the photocatalyst reaction were evaluated using the following equations.

1 $${\text{Degradation percentage}}:{ 1} - \, \left[ {{\text{C}}/{\text{C}}_{0} } \right] \, \times { 1}00$$

2 $${\text{Pseudo first order reaction kinetics}}: {\text{ln C}}_{0} /{\text{ C }} = {\text{ kt}}$$

where, C—Concentration of dye at time t , C_0_—Initial concentration of the dye at t = 0, k—Rate constant s^−1^.

### Electrochemical measurements

Mott-Schottky analysis was performed to confirm the conduction band value. Using 0.5 M Na_2_SO_4_ as an electrolyte solution, Ag/AgCl as the reference electrode, and platinum as the counter electrode with fixed frequency of 1 kHz. EIS analysis was carried out to determine the charge transfer ability of the catalyst using solution containing 5 mM [Fe (CN)_6_]^3-/4-^ and 0.1 M KCl. For electrode fabrication, 10 mg/mL of catalyst was uniformly dispersed using bath sonicator for 30 min. After sonication, 8µL of homogenous solution was drop casted on screen printed electrode (SPCE) and dried at room temperature.

## Results and Discussion

### Powder XRD results

**Figure 2:**
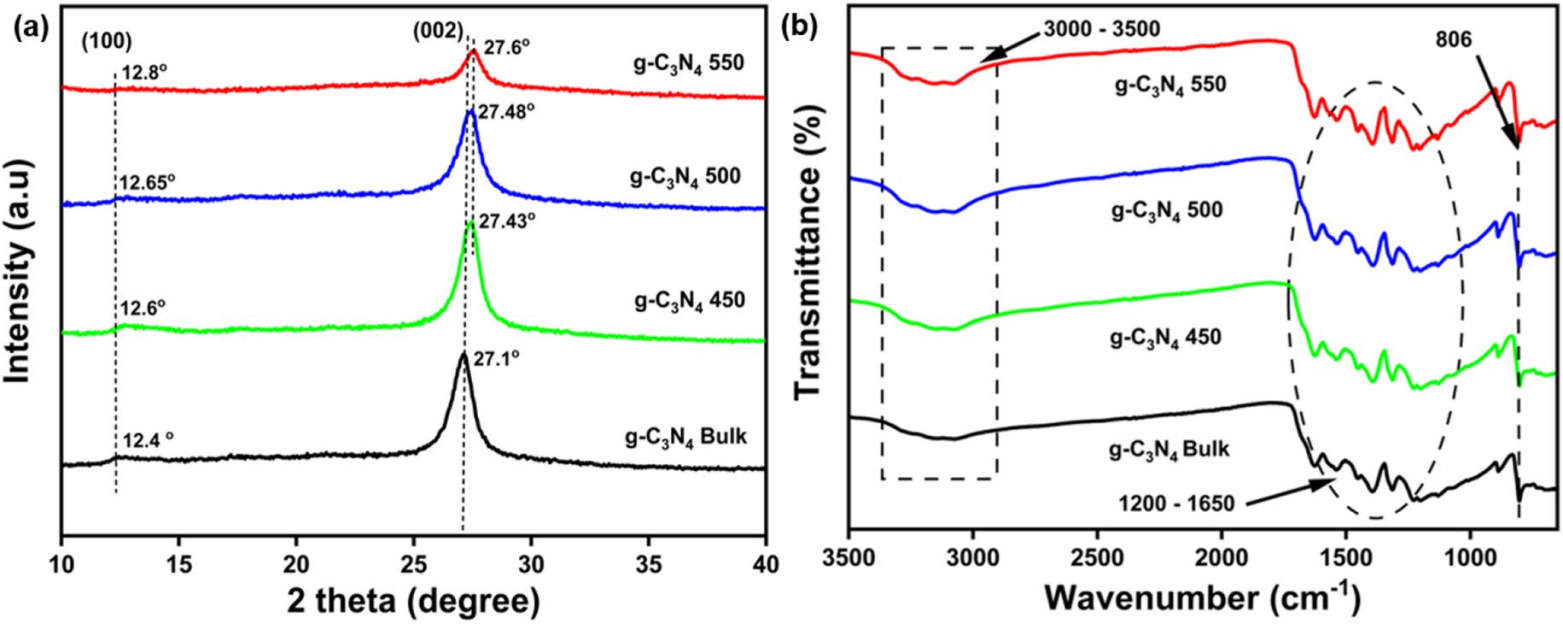
(**a**) Powder XRD results of g–C_3_N_4_ Bulk_,_ g–C_3_N_4_ 450, g–C_3_N_4_ 500, g–C_3_N_4_ 550, (**b**) FTIR spectra of the prepared samples.

Figure 2a showed the XRD pattern of all the prepared g–C_3_N_4_ samples. All the samples exhibited a similar XRD pattern that matches with JCPDS No. 87-1526, which corresponds to graphitic carbon nitride^47^. The diffraction pattern exhibited two prominent peaks at around ~ 27° (002) and ~ 12° (100), indicating the structural arrangement of tri-s-triazine heterocycles and the periodic repetition of heptazine units inside the plane, respectively. The thermally exfoliated samples showed broader peaks with reduced intensities when compared to their bulk counterparts, which probably depicted the reduced layer spacing in the exfoliated ones^48^. Thus, (002) peak strength of the samples steadily decreased, indicating a comparable exfoliation of the g–C_3_N_4_. The reduction in peak intensities, was associated with decrease in crystallinity with increase in surface area and porosity. The surface area and porosity are two key factors responsible for an efficient photocatalyst^49^. Moreover, higher angular shifts for the thermally exfoliated g–C_3_N_4_ samples [g–C_3_N_4_ 450 (12.6°) (27.43°), g–C_3_N_4_ 500 (12.65°) (27.48°), g–C_3_N_4_ 550 (12.8°) (27.6°)] when compared to the bulk one [g–C_3_N_4_ bulk (12.4°) (27.1°)] could again be attributed to the reduced interlayer spacing^50^. The crystallite size of the sample was calculated using Scherrer formula: D = Kλ/βcos θ, where D is crystallite size in (nm), β represents the full-width-at-half-maximum for the peak of the (101) plane, λ represents the wavelength of the X-ray radiation (0.15418 nm), and K is the Scherrer constant, which is assumed to have a value of 0.9^51^. The calculated crystallite size of the as prepared catalyst is listed in Table 1.

**Table 1:** Surface area, pore volume, crystallite size, band gap and, elemental composition of bulk and exfoliated g–C_3_N_4_.

Sample	Surface area m^2^/g	Pore volume (cm^3^/g)	Crystallite size (nm)	Band gap (eV)	Elemental composition	
					C (wt%)	N (wt%)
g–C_3_N_4_ Bulk	5.035	0.028	9.0	2.54	34.06	65.94
g–C_3_N_4_ 450	9.189	0.08	5.7	2.59	36.00	64.00
g–C_3_N_4_ 500	13.45	0.092	5.3	2.61	37.00	63.00
g–C_3_N_4_ 550	48.203	0.32	4.2	2.7	39.3	60.7

### FTIR analysis

Functional groups and chemical bonding of prepared materials were studied by Fourier transform infrared (FTIR) spectroscopy, as shown in Fig. 2b. Here all the prepared samples possess similar characteristics spectra of g–C_3_N_4_. The typical peaks attributed to g–C_3_N_4_ were present in all the samples prepared. The sharp peak observed at 806 cm^−1^ was attributed to the breathing mode of tri-s-triazine heterocycles. Moreover, the bands observed at 3000–3500 cm^−1^ depicted the stretching of O–H/N–H motifs in samples originating from the surface NH/NH_2_ groups and adsorbed water molecules^52,53^. In particular, the region from 1200 to 1650 cm^−1^ corresponds to the stretching vibrations of N–(C)_3_ and N=C heterocycles^54,55^. Finally, the similarity among the sample’s band stretching suggests that the thermal exfoliation does not make any significant changes in the g–C_3_N_4_^56^.

### XPS analysis

**Figure 3:**
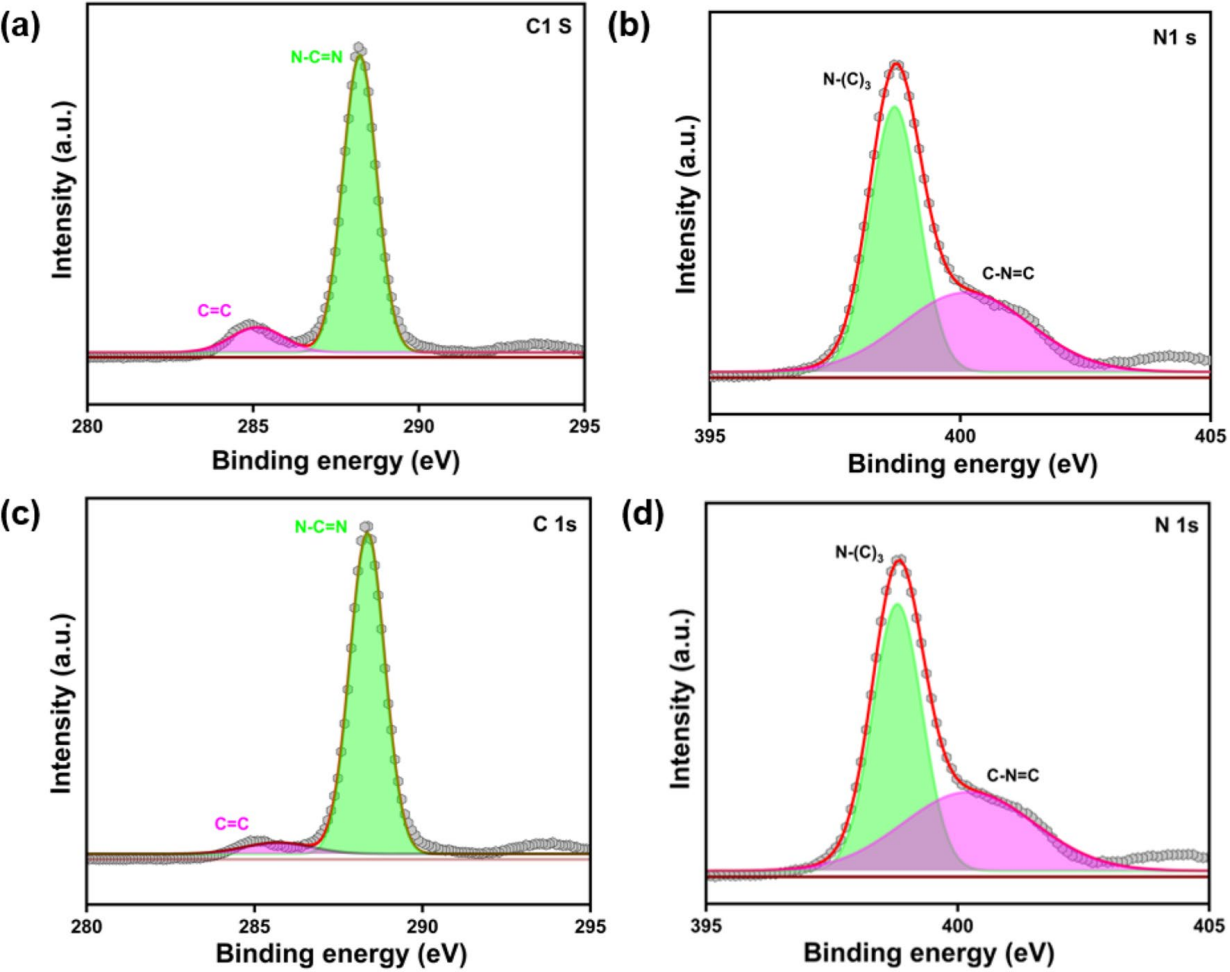
XPS spectrum of bulk g–C_3_N_4_ and g–C_3_N_4_ 550. (**a**) C1s spectra of bulk g–C_3_N_4_ (**b**) N1s spectra bulk g–C_3_N_4,_ (**c**) C1s spectra for g–C_3_N_4_ 550, (**d**) N1s spectra of g–C_3_N_4_ 550.

The XPS analysis was performed to analyze the detailed chemical status of the catalyst prepared. As observed from Fig. 3a, the C 1s spectrum of bulk g–C_3_N_4_ fitted into binding energies of 288.2 eV and 284.9 eV, which can be attributed to the graphitic sites present in the g–C_3_N_4_ matrix of SP^2^-hybridized carbon atom bonded to N in the aromatic ring (N–C=N), SP^2^ C=C bonds and C–NH_2_ bonds respectively. The spectra of bulk g-C_3_N_4_ N 1s in Fig. 3b also showed the fitted binding energy peaks of 398.7 eV and 400.15 eV which are attributed to the SP^2^ hybridized C=N–C bond in the triazine ring and N–(C)_3_ bonds ^57,58^. The XPS spectrum of g–C_3_N_4_ 550 C1 s and N1 s are shown in Figs. 3c, d. The (N–C=N) and (C=C) bonds’ binding energy peaks in the C 1s spectra were found at 288.34 eV and 285.341. N–(C)_3_ and C–N=C of N1s were attributed in the range of 398.86 eV and 401.1 eV which confirmed the triazine rings and SP^2^ hybridized carbon atom bonded N in the aromatic rings. This also confirmed that the TE–g–C_3_N_4_ had undergone no chemical structural changes in the presence of heat treatment. The survey scan spectrum of bulk g–C_3_N_4_ and g–C_3_N_4_ 550 are shown in Figure S1.

### Morphology analysis

**Figure 4:**
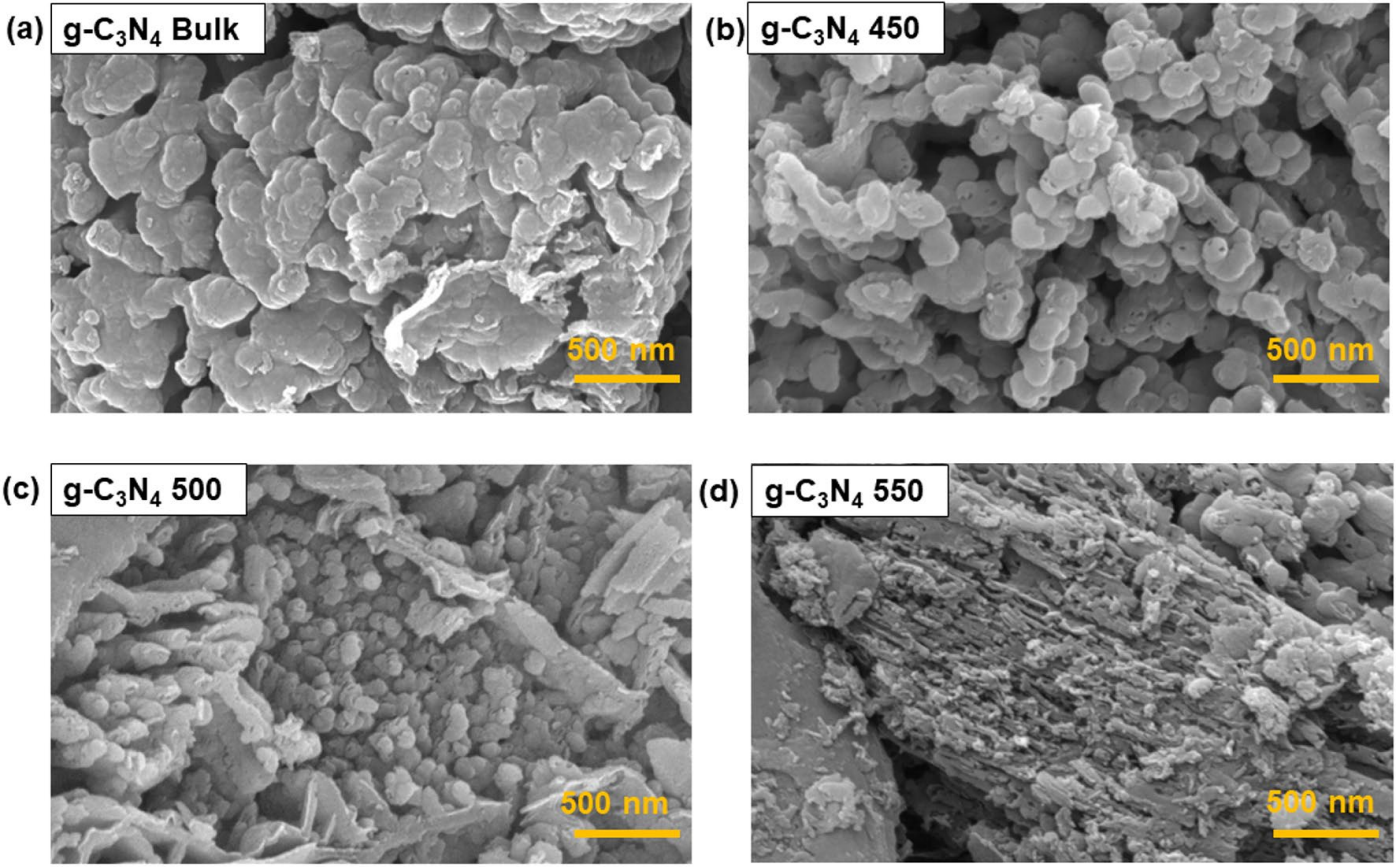
FESEM images of the synthesized samples: (**a**) g–C_3_N_4_ Bulk_,_ (**b**) g–C_3_N_4_ 450, (**c**) g–C_3_N_4_ 500, (**d**) g–C_3_N_4_ 550 photocatalyst.

Figure 4 illustrated the structural morphology of prepared samples which confirmed the effect of temperature on the thermal exfoliation of the prepared materials. In case of bulk g–C_3_N_4,_ flat, aggregated and non-porous flakes were observed while for exfoliated ones, thinner layers with rough surface and layered detachments were found with increasing exfoliation temperature as shown in Fig. 4b–d. There were similar reports in the literature for the exfoliated ones^59,60^. The EDAX analysis confirmed the presence of C and N and their respective percentages as shown in Figure S2. Elemental mapping of prepared samples shown in Figure S3 showed the uniform homogeneous distribution. Furthermore, the elemental composition of all prepared photocatalysts is listed in Table 1.

### Surface area analysis

**Figure 5:**
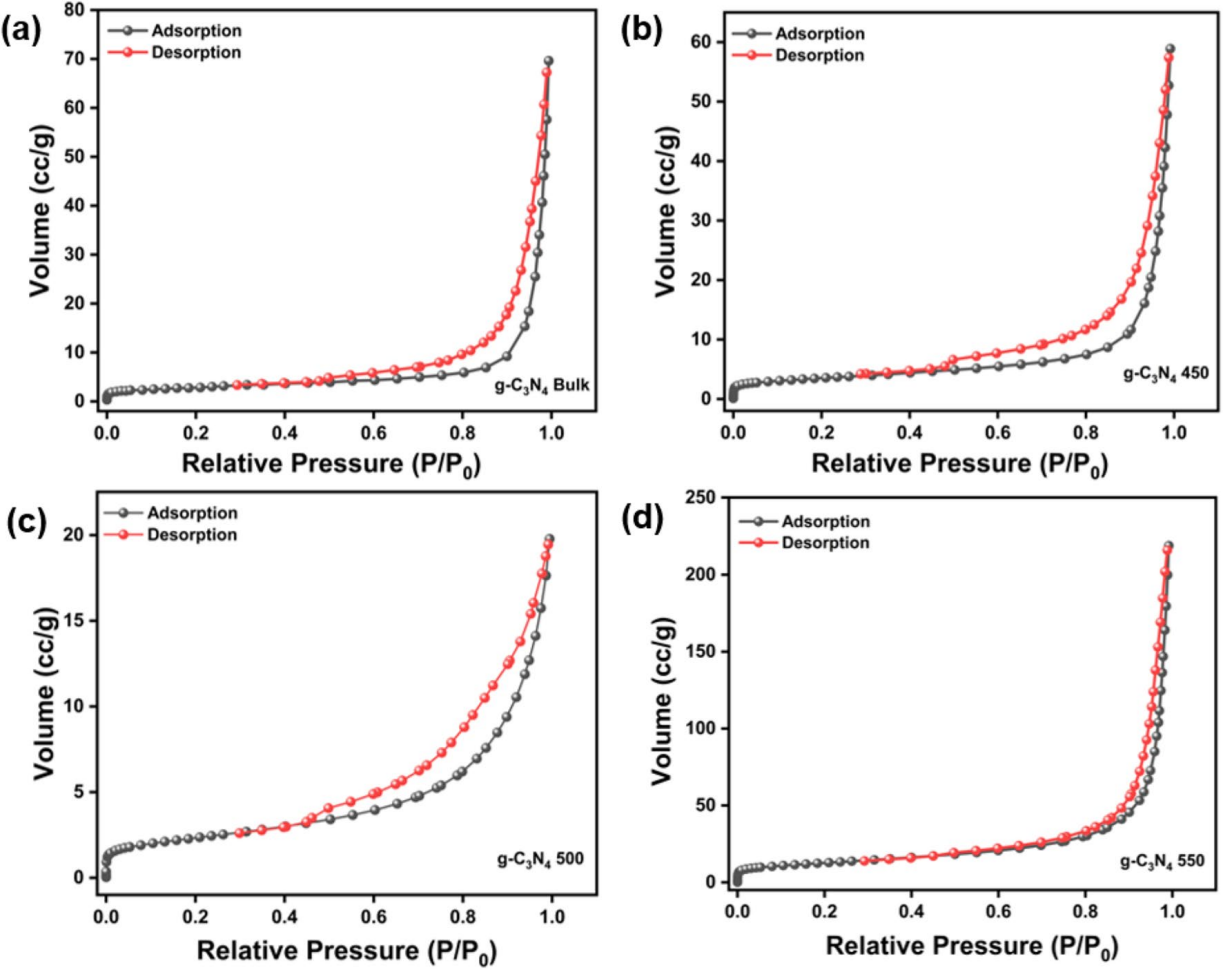
(**a**) N_2_ adsorption–desorption plots of (**a**) bulk g–C_3_N_4_ and, (**b**–**d**) exfoliated g–C_3_N_4_.

Figure 5 displays the N_2_ adsorption–desorption isotherms for the photocatalysts that were produced. The observed samples were determined to conform to the type IV isotherm and exhibited H3 hysteresis loop, consistent with findings described in the existing literature^61^. Moreover, it confirmed that all the prepared samples are multilayer—monolayer adsorption and are mesoporous materials. The specific surface area (S_BET_) and porosity for prepared samples increased with increasing thermal heat treatment temperature^47^. The specific surface area for g–C_3_N_4_ bulk_,_ g–C_3_N_4_ 450, g–C_3_N_4_ 500, g-C_3_N_4_ 550 are 5.035 m^2^/g, 9.189 m^2^/g, 13.45 m^2^/g and 48.203 m^2^/g with pore size distribution in the range of 2–20 nm (Figure S4). The increase in surface area upon increase in exfoliation temperature might be attributed to surface roughness, which was observed in FESEM analysis for the thermally exfoliated samples and it also goes well with XRD results. Furthermore, the specific surface area (S_BET_) and pore volume was also tabulated in Table 1.

### Optical study

**Figure 6:**
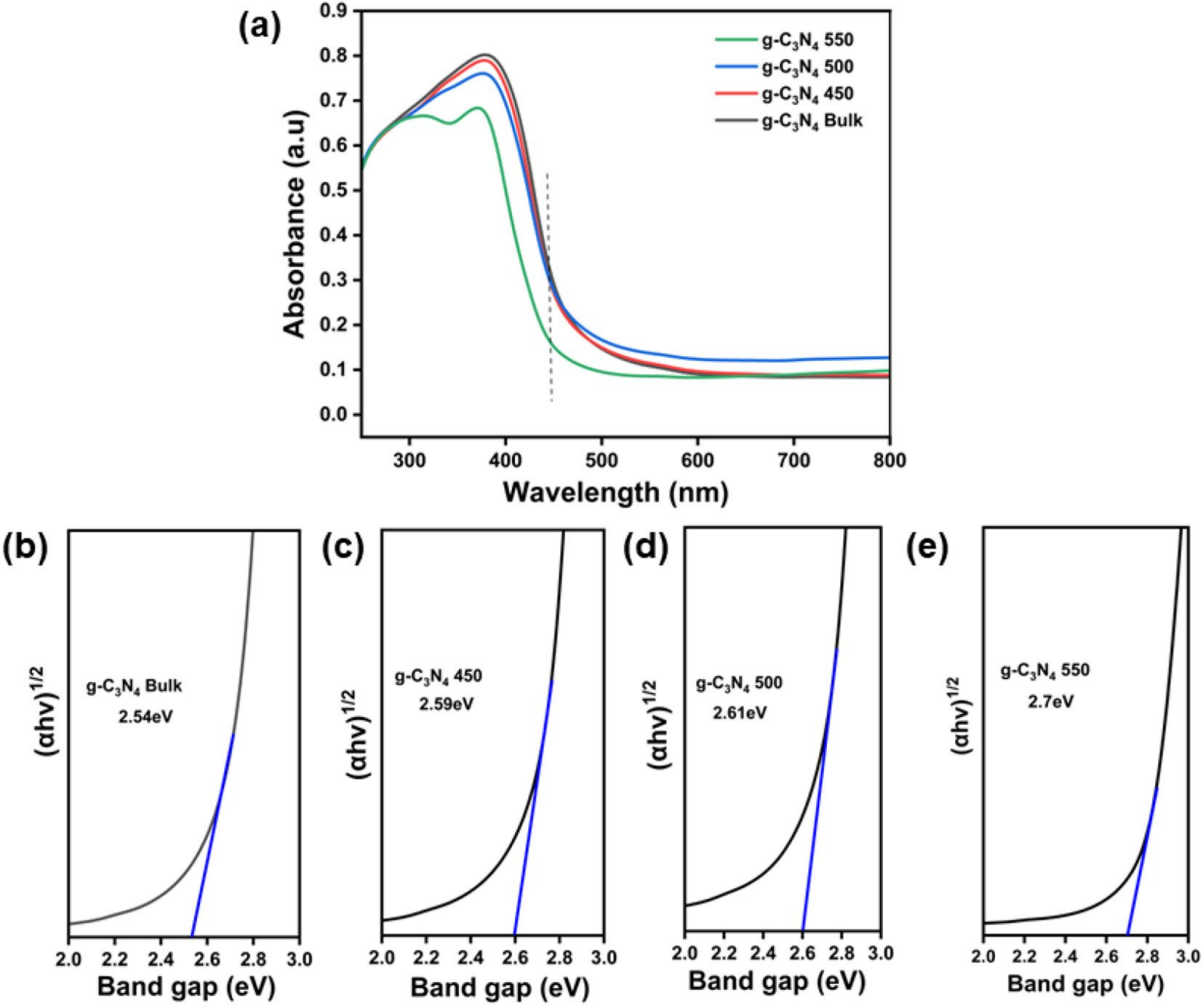
(**a**) UV–Visible absorbance spectra of g–C_3_N_4_ Bulk_,_ g–C_3_N_4_ 450, g–C_3_N_4_ 500 and g–C_3_N_4_ 550. (**b**–**e**) Band gap analysis-Tauc plot of the prepared samples.

The optical characteristics of the produced photocatalysts were examined using ultra-violet diffuse reflectance spectroscopy (UV-DRS). The optical absorption spectra of the prepared samples are shown in Fig. 6a. All the prepared samples exhibited absorption edge at 450 nm depicting intrinsic (π–π*) transition and showed blue shift after thermal exfoliation because of the quantum confinement effect^62,63^. Moreover, the band gap energies of the catalyst were determined using the tauc equation, and these values are illustrated in the Fig. 6b–e. The band gap energy of prepared samples increased with increasing thermal exfoliation temperature, which further confirmed the quantum confinement effect because of reduction in thickness of exfoliated photocatalysts^64^.

Furthermore, the band gaps of all photocatalysts are tabulated in Table 1. The band gap values were calculated using the following equation:

3 $${\text{Tauc plot}}: \alpha {\text{h}}\nu \, = {\text{ A }}\left( {{\text{h}}\nu \, - {\text{ Eg }}} \right)^{{{1}/{2}}}$$

where, α-absorption coefficient, A-Proportionality constant, h-Planck’s constant, ν-photons’ frequency, Eg-Bandgap.

### Photocatalytic activity

The photocatalytic performance of g–C_3_N_4_ bulk, g–C_3_N_4_ 450, g–C_3_N_4_ 500, and g–C_3_N_4_ 550 was assessed in relation to the degradation of organic dyes: MB, MO and, RhB, individually. Also, the adsorption efficiencies for both the bulk and exfoliated photocatalysts were studied in the absence of light. Figure S5 showed an increased adsorption efficiency for all the thermally exfoliated samples when compared to the bulk ones. This could be due to increased surface area for the exfoliated samples as confirmed by BET studies. Also, the photodegradation efficiency of prepared photocatalysts could be arranged in the following sequence: g–C_3_N_4_ bulk˂ g–C_3_N_4_ 450 ˂ g–C_3_N_4_ 500 ˂ g–C_3_N_4_ 550. The photodegradation efficiency of g–C_3_N_4_ 550 against MB, MO and RhB dyes were found to be 92 ± 0.18%, 93 ± 0.31%, and 95 ± 0.4% respectively within 60 min of UV light irradiation.

### Effect of catalyst

**Figure 7:**
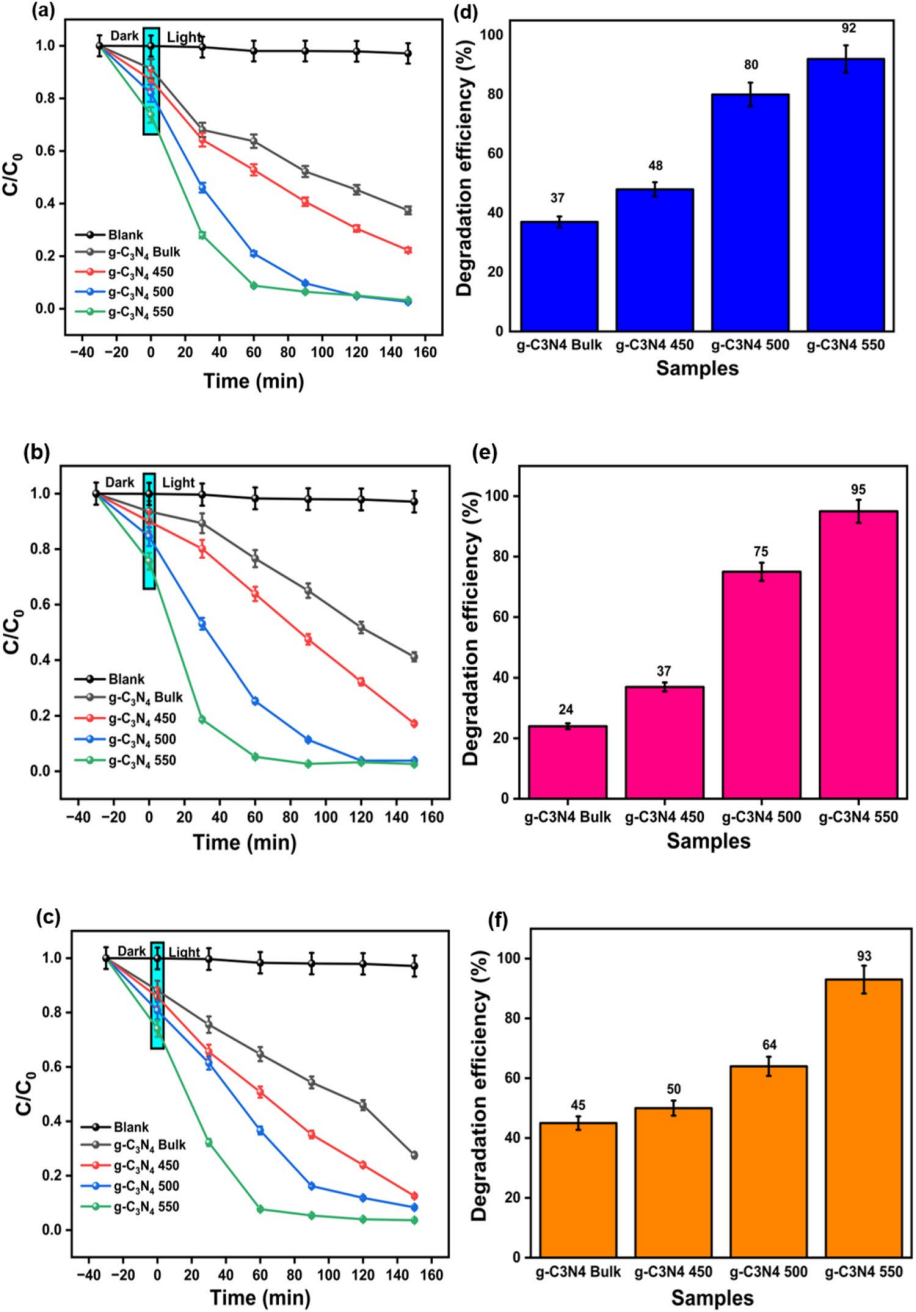
(**a**–**c**) Time dependent degradation kinetics measured as the concentration of dye at time t with respect to initial concentration (C/C_0_), (**d**–**f**) photocatalytic degradation efficiency of all the samples after 60 min of degradation.

The catalytic properties of the prepared catalysts were analyzed by performing photodegradation of all three dyes individually under irradiation of UV light. Initially, all the dyes were kept in the photocatalytic reaction chamber and irradiated for 60 min without a catalyst and their corresponding absorbance spectrum was recorded as shown in Fig. 7a–f. The blank test (black line in Fig. 7) showed no degradation of dyes. However, after addition of photocatalyst to the dye solutions, there was a remarkable reduction in the concentrations of all dye solutions. Figure 7a–c depicted the photocatalytic degradation of three dyes with irradiation time corresponding to the C/C_0_ in all prepared photocatalyst g–C_3_N_4_ bulk_,_ g–C_3_N_4_ 450, g–C_3_N_4_ 500, g–C_3_N_4_ 550. Figure 7d–f showed the degradation efficiency of all reported catalysts for all three dyes. The degradation kinetics of three dyes were fitted with first-order rate model. The rate constant values were calculated with the help of Eq. (2) mentioned in section “Photocatalytic experiments”.

Moreover, the rate constant for g–C_3_N_4_ 550 is higher in all three dye degradation experiments shown as shown in Table 2.

**Table 2:** Rate constant values for the bulk and exfoliated g-C_3_N_4_.

Photocatalyst	MB dye	RhB dye	MO dye
bulk g–C_3_N_4_	5.39 × 10^−3^ s^−1^	4.95 × 10^−3^ s^−1^	6.55 × 10^−3^ s^−1^
g–C_3_N_4_ 450	8.24 × 10^−3^ s^−1^	9.35 × 10^−3^ s^−1^	1.120 × 10^−2^ s^−1^
g–C_3_N_4_ 500	2.005 × 10^−2^ s^−1^	2.086 × 10^−2^ s^−1^	1.502 × 10^−2^ s^−1^
g–C_3_N_4_ 550	2.133 × 10^−2^ s^−1^	2.276 × 10^−2^ s^−1^	2.096 × 10^−2^ s^−1^

## Investigation of the photocatalytic reaction mechanisms

### Photoluminescence (PL) Spectroscopy and EIS analysis

**Figure 8:**
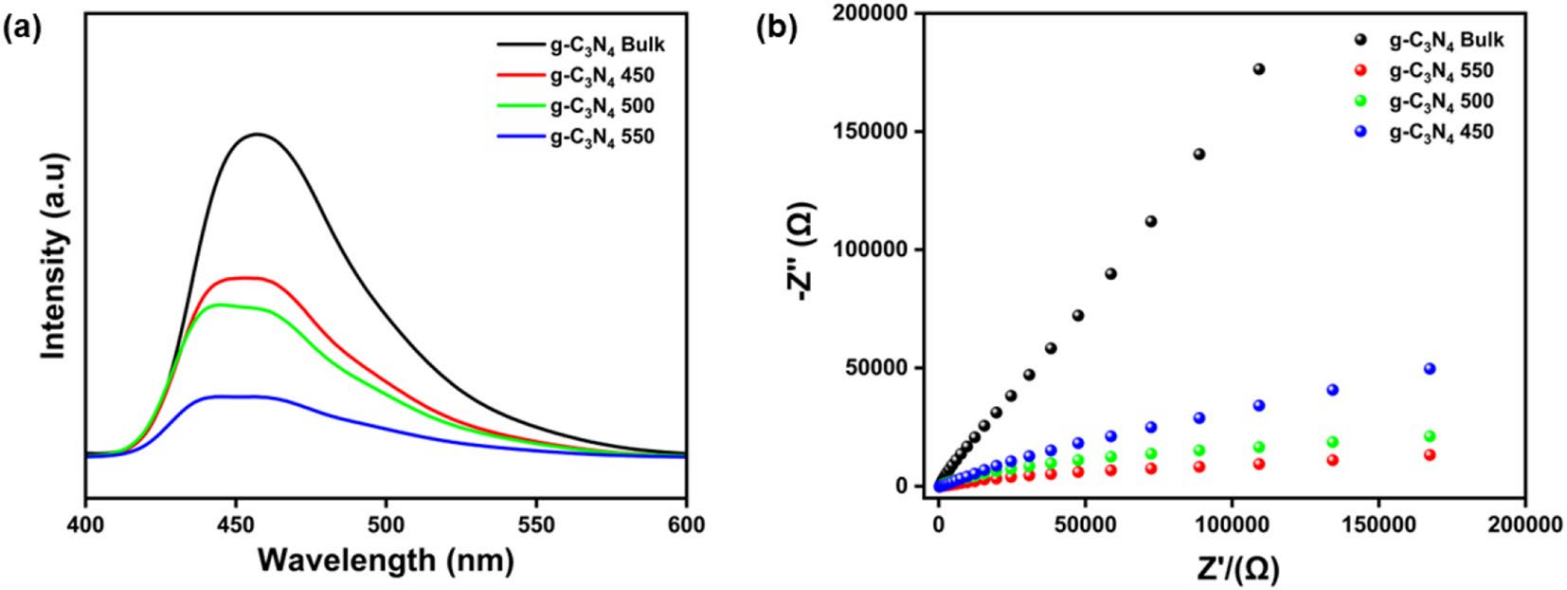
(**a**) Photoluminescence (PL) spectra of prepared photocatalysts, (**b**) EIS analysis of the prepared photocatalysts.

A good photocatalyst are expected to have high charge transfer rate and low recombination rate of photoexcited holes and electrons^42,65^. PL study and EIS Nyquist plot were used to investigate the catalyst charge carrier recombination effect and charge transfer resistance. PL study was performed to find the recombination rate of photogenerated electron–hole pair of the prepared materials. A wavelength of 400–600 nm was used to examine the emission spectrum of the bulk and exfoliated g–C_3_N_4_. Figure 8a showed the PL spectra of all catalysts and the peak intensity revealed the recombination rate of photoexcited holes and electrons. In all the catalysts, the peak at 450 nm confirmed the π–π* transitions^66^. The lower intensity for g–C_3_N_4_ 550 indicated the low electron–hole recombination rate, Moreover, all the thermally exfoliated samples showed significantly decreased intensity compared with g–C_3_N_4_ bulk, confirming the suppression of charge recombination in thermally exfoliated samples. The charge transfer resistance kinetics was also investigated by EIS analysis. Figure 8b showed the EIS Nyquist plots in which g–C_3_N_4_ 550 exhibited a much smaller arc radius suggesting a higher charge transfer rate than g–C_3_N_4_ bulk and other thermally exfoliated samples.

### Radical scavenging tests

**Figure 9:**
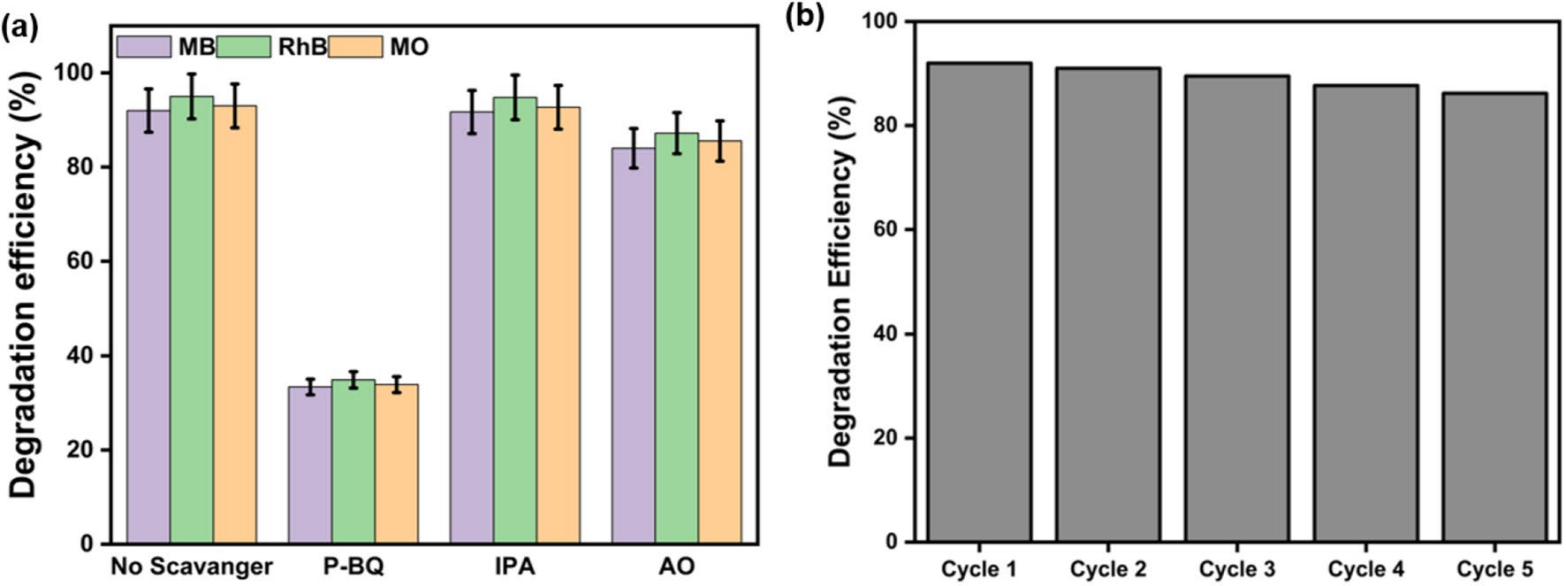
(**a**) Radical scavenging study to determine the reaction mechanisms for g–C_3_N_4_ 550 catalysts against three textile dyes. Superoxide anions, hydroxyl radicals and photoinduced hole are quenched by para-benzoquinone (P–BQ), isopropyl alcohol (IPA) and ammonium oxalate (AO), respectively. (**b**) Reusability analysis of g–C_3_N_4_ 550 catalysts for five successive cycles.

In a typical photocatalytic degradation process, the photo-induced hole (h^+^), hydroxyl radical (^.^OH), and superoxide radical (^.^O_2_^–^) played a vital role in the degradation of organic pollutants. Thus, it is imperative to identify the most reactive species that enabled the degradation of dyes. As shown in Fig. 9a, without the addition of any scavengers, there was no significant change in the degradation efficiency for all the three dyes. However, in the presence of scavenger para-benzoquinone (P–BQ), there was a significant drop in the degradation efficiency, indicating the superoxide radical to be very important for the degradation of the dyes. Next to P–BQ, there was a slight reduction in the degradation efficiency for all dyes in case of AO (*p* = 0.0038) which indicated that the photoinduced holes also contributed to the catalytic reaction but in a minimal way. There was no significant change (*p* = 0.8338) in the degradation efficiency upon addition of IPA, indicating the poor contribution of hydroxyl radicals towards the photocatalytic reaction. This also goes well with reported literature citing the fact that the valance band edge around + 1.8 eV of g–C_3_N_4_ is energetically unfulfilled for generation of ^.^OH^67^. To further confirm the same and to understand the nature of active species involved in the photodegradation, the band position of each catalyst was measured. The positions of the conduction band (CB) and valance band (VB) of the prepared catalyst were determined, and listed in Table 3 using the following empirical formula:

4 $${\text{E}}_{{{\text{CB}}}} = X {-}{\text{ E}}^{{\text{e}}} - \, 0.{\text{5 E}}_{{\text{g}}}$$

5 $${\text{E}}_{{{\text{VB}}}} = {\text{E}}_{{{\text{CB}}}} + {\text{E}}_{{\text{g}}}$$

where, *X*—absolute electronegativity of the material. For g–C_3_N_4_ is 4.72 eV^68^. E^e^—energy of free electrons on the hydrogen scale (E^e^ = 4.5 eV), E_CB_—conduction band potential, E_VB_—valance band potential.

**Table 3:** Band gap (E_g_) and band potentials (E_CB_, E_VB_) of TE- g-C_3_N_4_.

Photocatalyst	Band gap (eV)	Conduction band potential (eV) [E_CB_]	Valance band potential (eV) [E_VB_]
bulk g–C_3_N_4_	2.54	− 1.05	1.49
g–C_3_N_4_ 450	2.59	− 1.075	1.51
g–C_3_N_4_ 500	2.61	− 1.085	1.52
g–C_3_N_4_ 550	2.7	− 1.13	1.57

**Figure 10:**
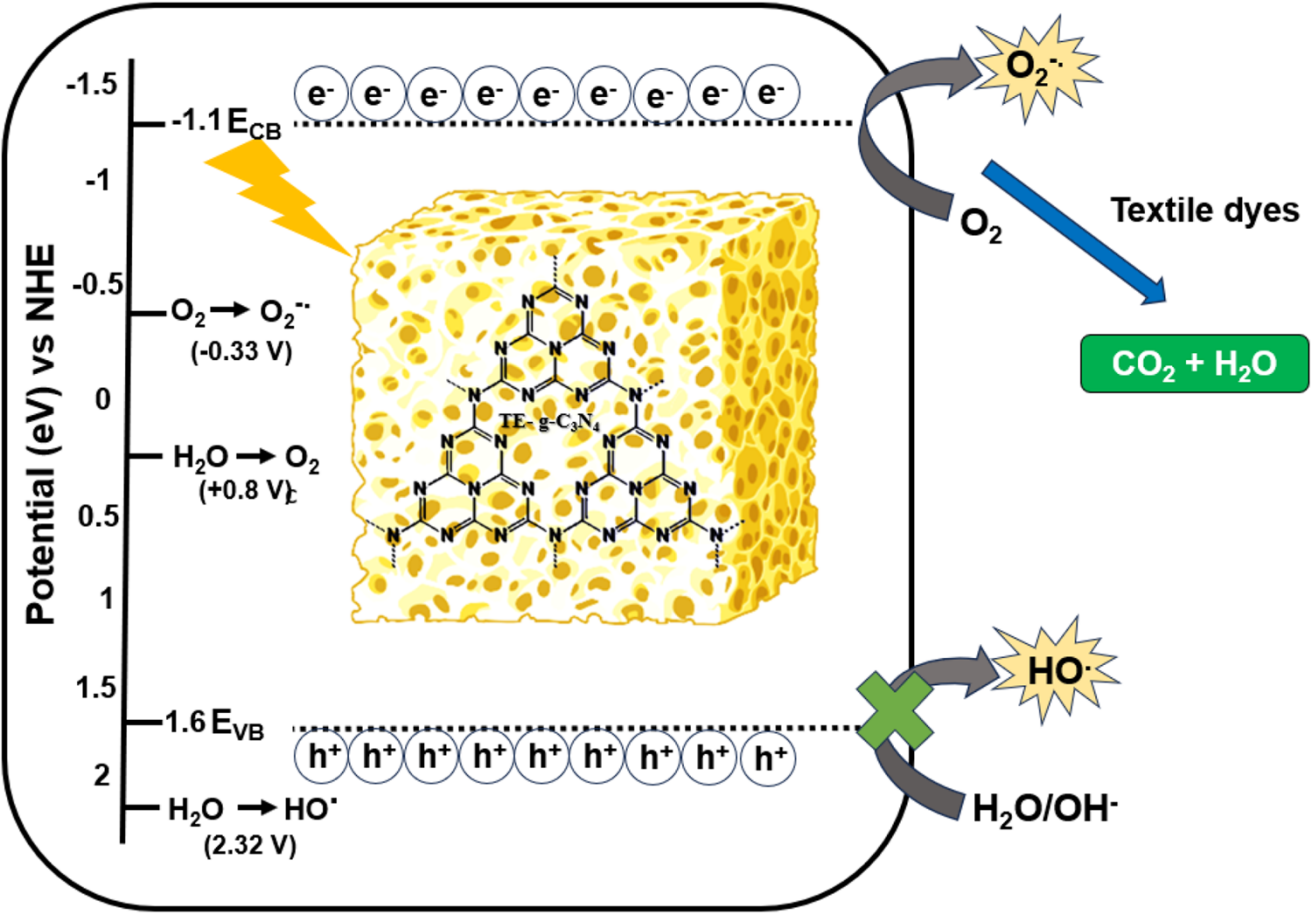
The proposed photocatalytic reaction mechanism of TE–g–C_3_N_4_ against textile dye pollutants.

Additionally, Mott-Schottky analysis was also performed for the best catalyst g-C_3_N_4_ 550, which were used for the scavenging study to confirm the conduction band potential (E_CB_). The flat band potential (V_fb_) for the catalyst was calculated as − 1.21 eV using the Mott-Schottky plot with respect to Ag/AgCl as reference electrode as shown in Figure S6. In general, for n-type semiconductor catalyst V_fb_ is ∼ 0.1 eV more negative than E_CB_^69^. Thus, E_CB_ for g–C_3_N_4_ was calculated to be ∼ − 1.1 eV, and E_VB_ was found to be 1.6 eV. This result agreed with the results from the reported literature^67,70^. The proposed photocatalytic mechanism of TE–g–C_3_N_4_was illustrated in the Fig. 10.

### Evaluation of the reusability of the photocatalysts

The best-performing g–C_3_N_4_ 550 was tested for its stability and reusability for five continuous cycles with MB dye. Figure 9b showed the results for the reusability cycle experiments of the photocatalysts. It was interesting to find that only very slight decline in the degradation efficiency of the photocatalyst after 5 continuous cycles from around 92–86.2%. This slight decline might also be due to the loss of photocatalytic materials due to centrifugation process that was collected after each cycle. Additionally, post reusability of g–C_3_N_4_ 550 catalysts was analysed with XRD and FTIR which did not show any significant changes in in the spectra as shown in Figure S7. This test confirmed the reusability of prepared photocatalysts, which was very important for translating this technology to more practical applications.

## Conclusion

The TE–g–C_3_N_4_ was synthesized by subjecting the bulk g–C_3_N_4_ to high temperatures and found that g–C_3_N_4_ exfoliated at 550 °C showed better photocatalytic degradation efficiency in comparison with its bulk counterparts as well as catalysts synthesized at other lower temperatures. TE–g–C_3_N_4_ were observed to be porous and have rough surfaces resulting in high surface area as measured using BET, and SEM analysis. Due to increased specific surface area (48.203 m^2^/g), and band gap (2.7 eV), the results noticed for g–C_3_N_4_ 550 against MB, MO and RhB dyes were found to be 92 ± 0.18%, 93 ± 0.31%, and 95 ± 0.4% respectively in 60 min of light irradiation and all these results followed first-order rate kinetics. In a radical scavenging study, it was found that the superoxide radicals played a crucial role in the photocatalytic degradation reaction followed by photogenerated holes in a very minimal way. However, further investigations on the in-depth mechanisms of how holes contribute to the degradation of dyes are warranted. Furthermore, the EIS and PL study confirmed the charge transfer ability and carrier recombination effect which further confirmed that the efficiency of the catalyst improved in the following sequence: g–C_3_N_4_ bulk ˂ g–C_3_N_4_ 450 ˂ g–C_3_N_4_ 500 ˂ g–C_3_N_4_ 550. The reusability studies on the best performing photocatalyst confirmed its excellent reusability with minor decrease in activity from 92 to 86.2% after 5 consecutive runs. Thus, TE–g–C_3_N_4_ could be a potential green photocatalyst which could be deployed in the wastewater treatment applications after a few further pilot scale investigations.

### Supplementary Information

Supplementary Figures.

## Data Availability

All data that supports the findings of the study are available from the corresponding author on reasonable request.
