# Supplementary material for: Efficient photocatalytic degradation of textile dye pollutants using thermally exfoliated graphitic carbon nitride (TE–g–C3N4)
Source: Sci Rep. 2024 Jan 27;14:2284. doi: 10.1038/s41598-024-52688-y (PMC10821873; doi:10.1038/s41598-024-52688-y)
Supplement: Supplementary file 1 — Supplementary Figures. [file 41598_2024_52688_MOESM1_ESM.pdf]

Supplementary information

Efficient photocatalytic degradation of textile dye pollutants using thermally exfoliated graphitic carbon nitride (TE- g-C_3N_4)

Selvaganapathy Ganesan[1,3], Thangavelu Kokulnathan[2], Shanmugam Sumathi[1] & Arunkumar Palaniappan[3, *]

[1]Department of Chemistry, School of Advanced Sciences, Vellore Institute of Technology, Vellore 632014, Tamil Nadu, India.
[2]Department of Electro-Optical Engineering, National Taipei University of Technology, Taipei 106, Taiwan.
[3]Centre for Biomaterials, Cellular and Molecular Theranostics (CBCMT), Vellore Institute of Technology, Vellore 632014, Tamil Nadu, India.

*Corresponding author:

Dr Arunkumar Palaniappan,
Assistant Professor,
Centre for Biomaterials, Cellular and Molecular Theranostics (CBCMT),
Vellore Institute of Technology, Vellore 632014, Tamil Nadu, India.
arunkumar.p@vit.ac.in

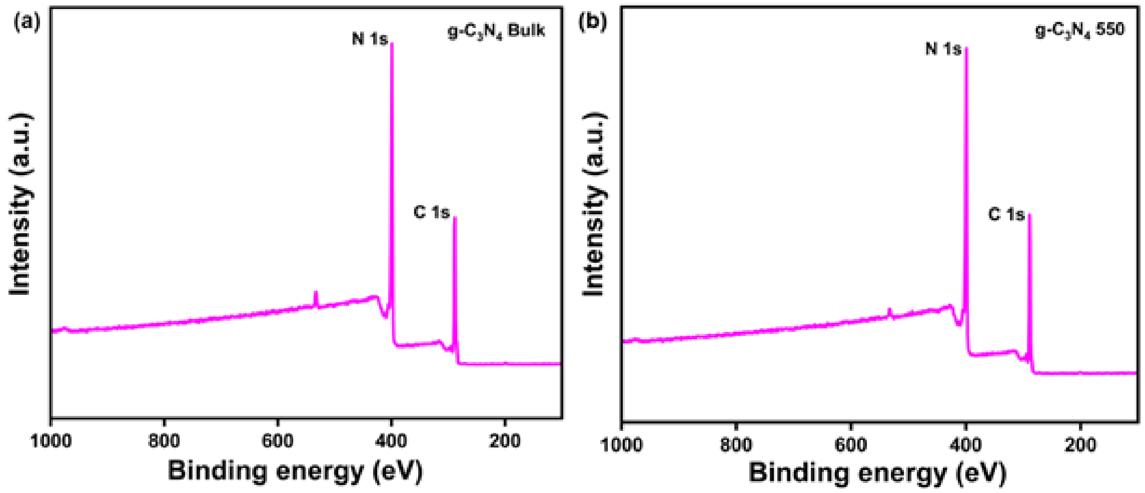

Figure S1. (A) XPS survey scan spectrum of g-C_3N_4 bulk **(b)** survey scan spectrum of g-C_3N_4 550

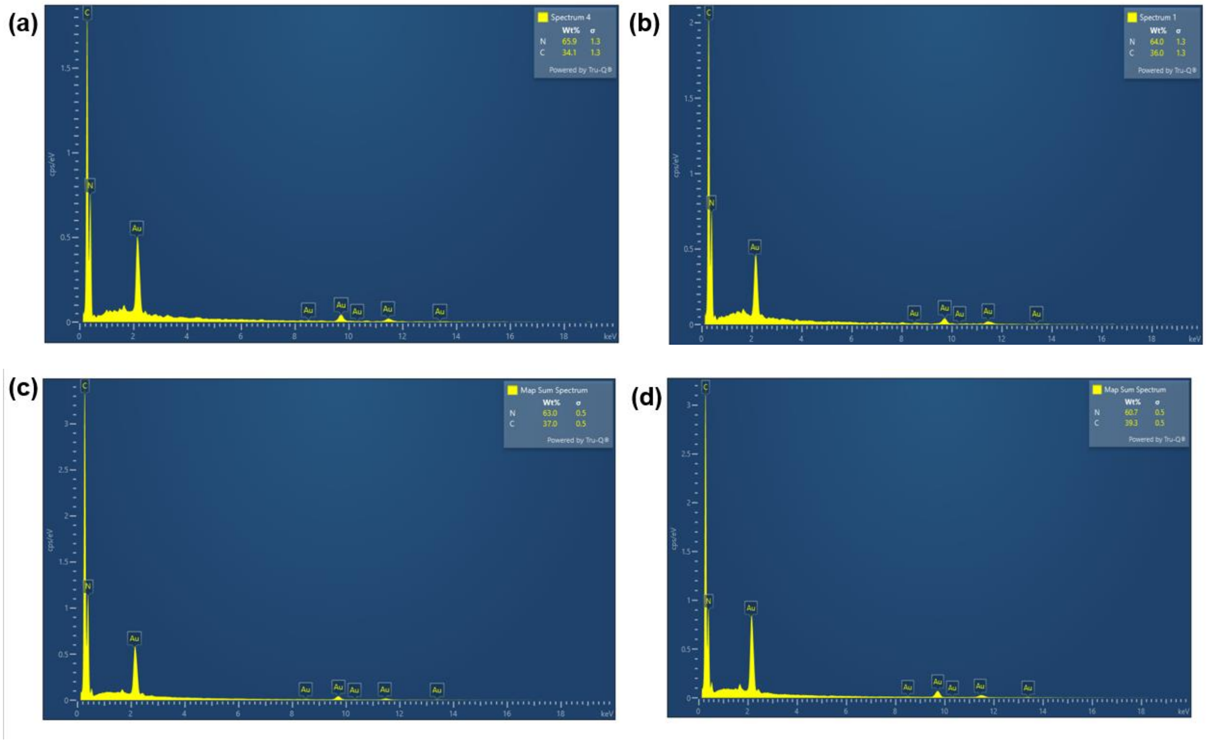

Figure S2. (a-d) EDAX spectrum of g-C_3N_4 bulk, g-C_3N_4 450, g-C_3N_4 500, g-C_3N_4 550

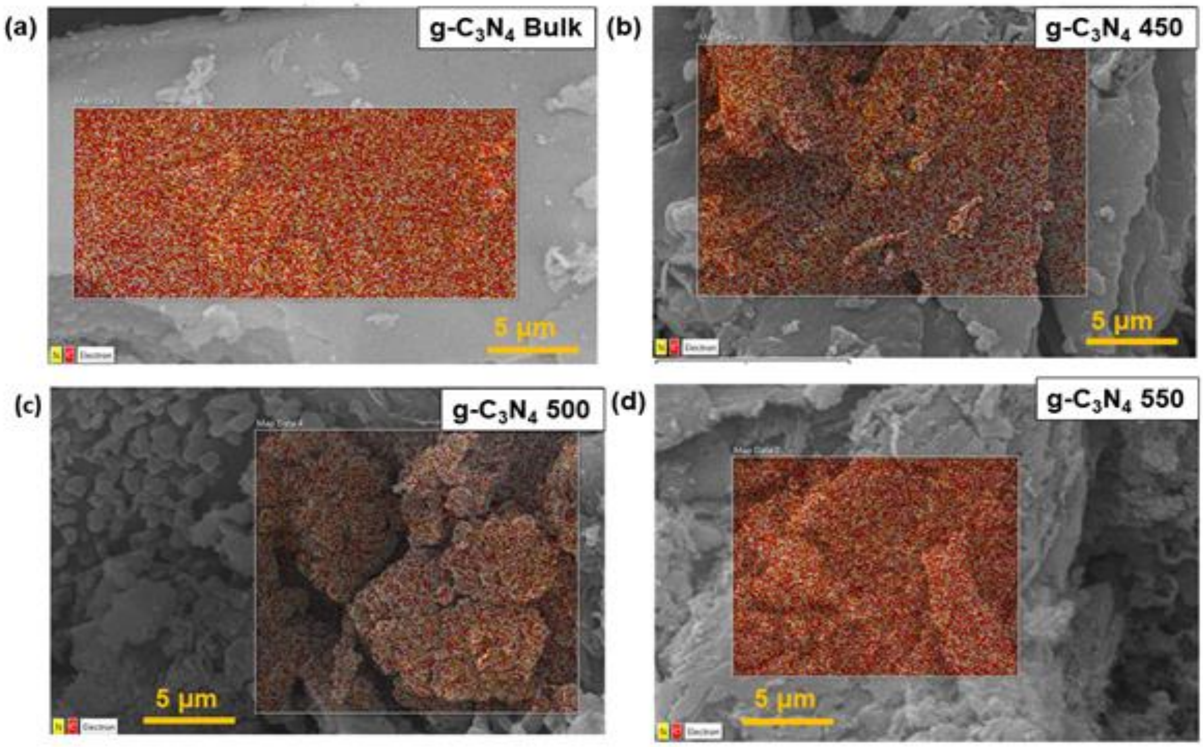

Figure S3. (a) Elemental mapping of bulk g-C_3N_4 and, (b-d) exfoliated g-C_3N_4

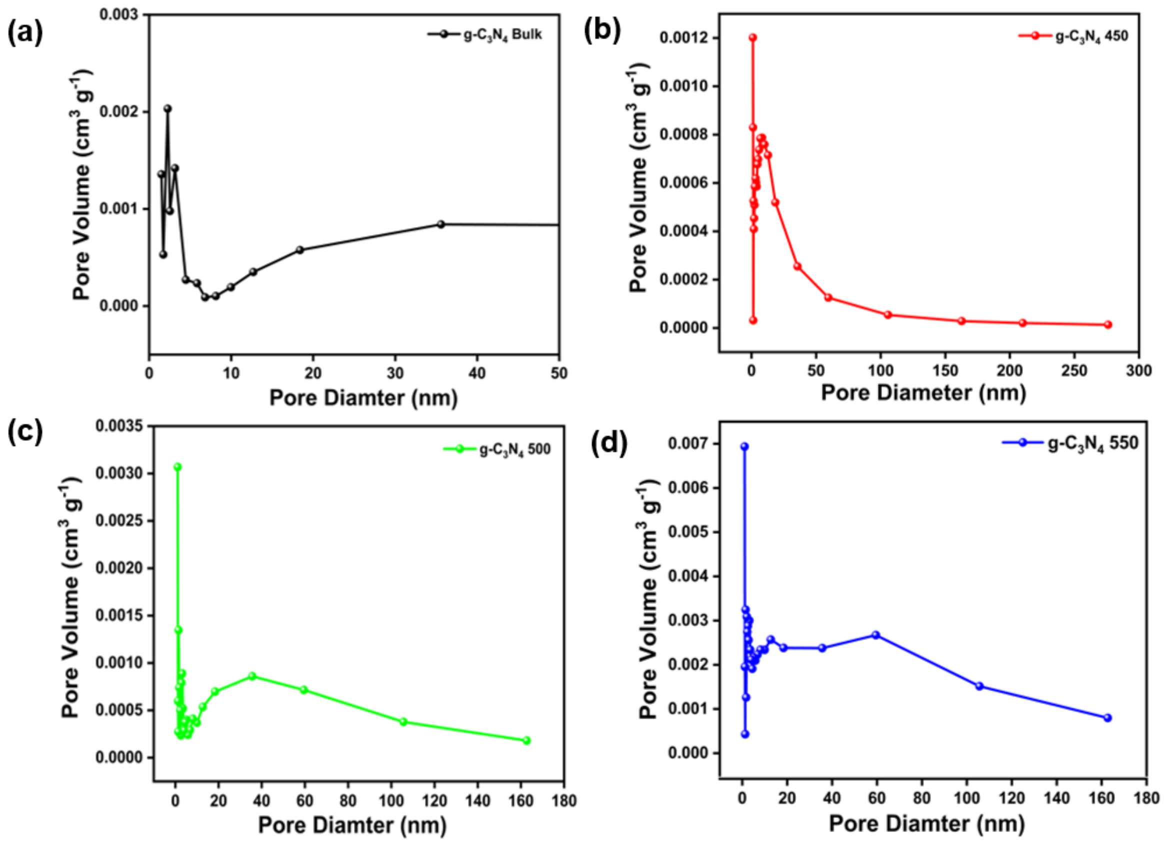

Figure S4. (a-d) BJH pore size distribution of g-C_3N_4 bulk and exfoliated g-C_3N_4

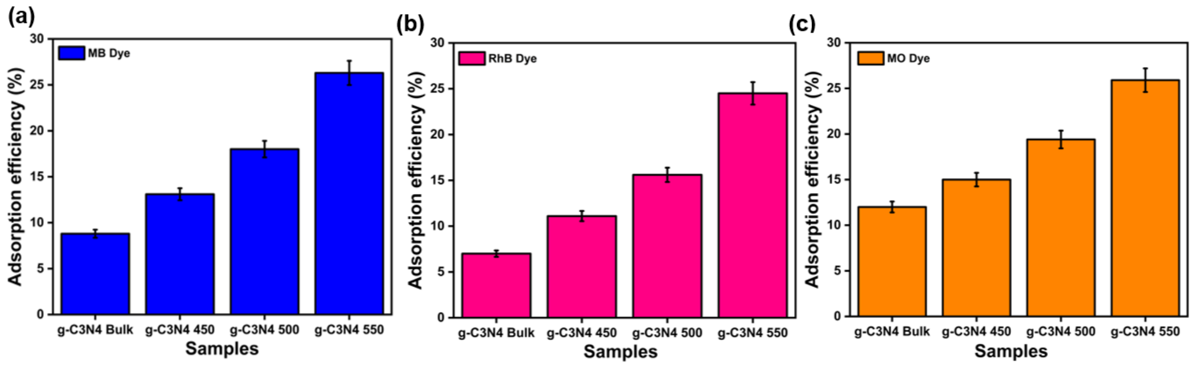

Figure S5. (a-c) Adsorption efficiency of bulk and exfoliated g-C_3N_4 Photocatalyst against three different dyes.

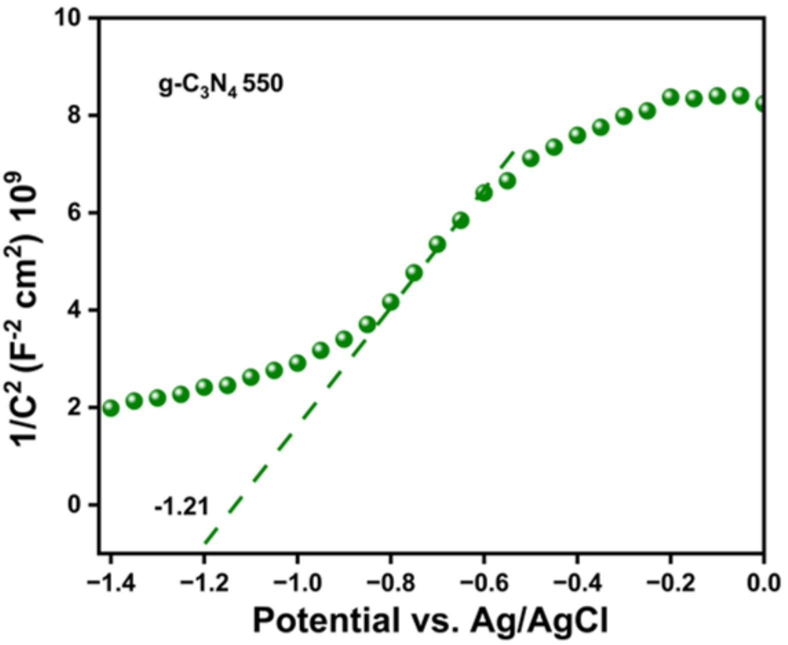

Figure S6. Mott-Schottky plot for g-C_3N_4 550.

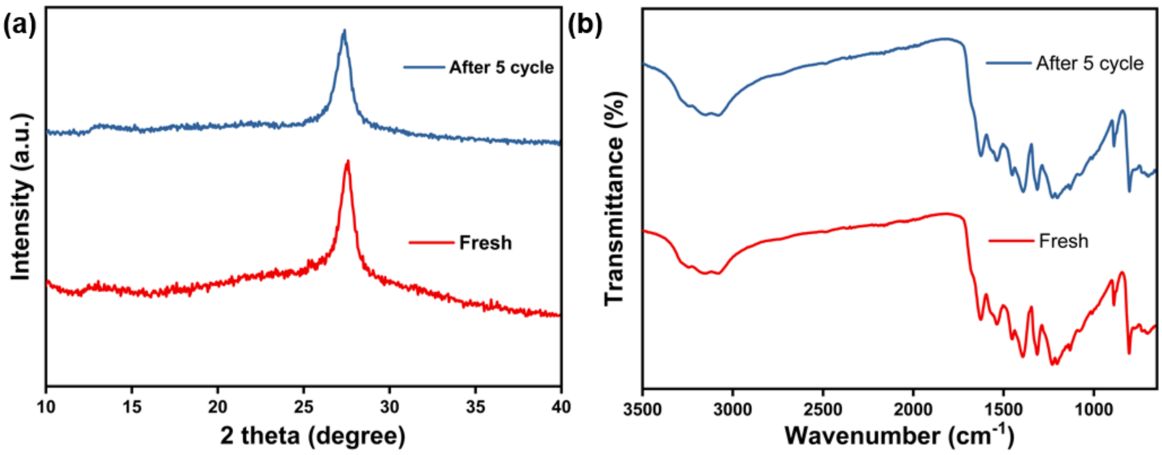

Figure S7. (a) XRD, (b) FTIR spectra of g-C_3N_4 550 post reusability study.
